# Canada’s role in strengthening global health security during the COVID-19 pandemic

**DOI:** 10.1186/s41256-020-00146-3

**Published:** 2020-04-20

**Authors:** Vijay Kumar Chattu, Anil Adisesh, Sanni Yaya

**Affiliations:** 1 Division of Occupational Medicine, Department of Medicine, University of Toronto, Toronto, ON Canada; 2 Occupational Medicine Clinic, St. Michael’s Hospital, Unity Health Toronto, Toronto, ON Canada; 3 Institute of International Relations, The University of the West Indies, St. Augustine, Trinidad and Tobago; 4 School of International Development and Global Studies, Faculty of Social Sciences, University of Ottawa, Ottawa, ON Canada; 5 The George Institute for Global Health, University of Oxford, Oxford, UK

**Keywords:** Coronavirus outbreak, COVID-19, Pandemic, Epidemic, Global health security, Canada, Public health, Epidemic curve, Public health emergency of international concern

## Abstract

The world is confronted by the current pandemic of Corona Virus Disease (COVID-19), which is a wake-up call for all nations irrespective of their development status or geographical location. Since the start of the century we have seen five big infectious outbreaks which proved that epidemics are no more regarded as historic and geographically confined threats. The Canadian government underlined that these infectious disease outbreaks are threats to global health security and disrupt societal wellbeing and development. In this context, the Public Health Agency of Canada is proactive and has shown its preparedness for outbreaks of emerging and epidemic-prone diseases, and in dealing with these pathogens. Even before the declaration of pandemic, Canada has proved its global health leadership by ensuring collective action and multisectoral coordination which still remains a serious challenge especially for low and middle- income countries with existing poor health systems. In this article we discuss how Canada is addressing the global challenges posed by the COVID-19 pandemic through its leadership and practice of global health diplomacy.

## Background

On March 11, 2020 the World Health Organisation (WHO) declared COVID-19 a pandemic after the virus affected more than 118,000 people in 114 countries and taking the lives of 4291 in the epidemic. Only a few weeks earlier on January 30, 2020, WHO had declared the COVID-19 outbreak a Public Health Emergency of International Concern (PHEIC) [1]. As of April 15, 2020, the WHO situation analysis of COVID-19 reported 1,914,916 confirmed cases from 210 countries and territories with 56,985 deaths [2]. In Canada, as of April 15, 2020 there were 27,557 confirmed COVID-19 cases with 954 reported deaths [3]. The current COVID-19 is a rapidly evolving global challenge and like any pandemic, it weakens health systems, costs lives and also poses great risks to the global economy and security. According to estimates by Bloomberg business, COVID-19 could cost the global economy $2.7 trillion equivalent to the entire GDP of the UK [4]. The International Air Transport Association (IATA) sees that the global losses for the air transport industry could be over $ 113 billion dollars [5]. According to WHO, Global public health security is defined as “the activities required to minimize the danger and impact of acute public health events that endanger the collective health of populations living across geographical regions and international boundaries.” [6]. In this context, Canada is doing its best through its public health system to deliver appropriate and evidence-based response measures to tackle this global public health threat. This commentary discusses how Canada is responding to this pandemic in promoting global health security by engaging with various international and global stakeholders through the practice of global health diplomacy.

### Canada’s public health leadership and its practice of global health diplomacy

The Government of Canada has been working with international partners, provinces and other stakeholders since the beginning of the COVID-19 epidemic. The Public Health System of Canada is well prepared to address the COVID-19 outbreak while working with various international partners including WHO. This commitment with high level intersectoral collaboration has resulted in dedicated funding of $52.6 million given for 96 research teams to conduct research with collaborating institutions in Africa and Asia to study and enhance the clinical and public health responses and to improve global health security [7]. As described by Kickbusch et al., Global Health Diplomacy aims to capture the multi-level and multi-actor negotiation process that shapes and manages the global policy environment for health [8]. The Canadian government has engaged in global health diplomacy with G7 (Group-7) finance ministers, multilateral organizations such as WHO, international health regulators (United States Federal Drugs Administration and European Medicines Agency) and bilateral partners at multiple levels to mobilize the resources for containing the pandemic. Thus, Canada has emphasized and demonstrated how collective international public health action can build a safer future for humanity which is the ultimate goal of global public health security. A summary of the activities by the Canadian government is shown below. (Table 1).

**Table 1:** Canada’s financial commitments and assistance in response to the COVID-19 epidemic

Level	Purpose	$ in million (CAD)	Activities/ Services provided
International and / Global	International Assistance	50	To strengthen the health systems and to improve early case detection
	Support to WHO	2	To assist vulnerable countries
	Material support	N/A	Supplied 16 tons of personal protective equipment to China
	Ensuring global health security by G7 Health and Finance ministers	N/A	Discussion and sharing responses and approaches to protect health of global population
	Collaboration with US Food and Drug Administration (US-FDA) and European Medicines Agency (EMA)	N/A	To support and coordinate rapid regulatory responses for vaccines and medicines
	Research	275	Vaccine and Antiviral development and clinical trials including in Canada. The fund has a global scope in recognition of the needs of affected LMICs.

Source: Government of Canada, Coronavirus disease (COVID-19): Canada’s response

### Strategies for strengthening global health security

The pandemic goals are first to minimize serious illness and overall deaths and secondly to minimize societal disruption and mitigate community transmission. According to recent statistics, Canada ranks number five (5) in the Global Health Security (GSI) Index which is the first comprehensive assessment and benchmarking of health security and related capabilities across 195 countries that make up the State Parties to the WHO’s International Health Regulations (IHR), 2005 [9]. As part of strengthening surveillance systems, the Public Health Agency of Canada ensures meeting the requirements for IHR and reports to WHO within 24 h its assessment of public health information. As of March 18, 2020, Canada has implemented a ban on all foreign nationals entering Canada (by air or marine) and also imposed a 30-day restriction on all non-essential travel at the Canada- U.S border. The Government’s Travel advice and advisories warrants all persons to avoid all non- essential international travel including to United States with an aim to minimize the risk of exposure to COVID-19 in Canada. In this context, the Act/ Regulation came into force from 26 March, 2020 which prohibits entry into Canada from U.S (effective until 21 April 2020) and prohibition of entry into Canada from any country other than US (until 30, June 2020) to support its continued focus on reducing the introduction and further spread of COVID-19.

Overall Canada has strengthened its case detection, testing (providing real time reverse transcription-polymerase chain reaction test), infection prevention and control. In addition, the government is also working with international regulators to help fast-track clinical trials and applications for vaccines, treatments and diagnostic tests. All these measures for inbound and outbound surveillance are very critical in addressing the pandemic as about 71% of all COVID-19 cases were exposed in the community while the remaining 29% were either exposed while travelling or exposed to a traveler returning to Canada [3]. The public health measures are targeted to “flatten the curve” thereby slowing acceleration of the number of cases in a community, reducing the peak number of cases during the pandemic and related health care demands on hospitals and infrastructure, and decreasing overall cases and health effects [10].

### Coping with the challenges of global supply chain dynamics

The growing interdependence of the world’s economies by cross-border trade in goods, services, technology and flows of investment, information and people is critical in this globalized world. However, the COVID-19 pandemic has posed many challenges to many economies (developing and developed) worldwide which has created more barriers for free trade, restricted commercial activities and disrupted the positive spirit (e.g. the President of U.S asked the medical supply firm 3M to stop selling N95 respirators to Canada and Latin American markets by invoking the Defence Production Act in order to focus on domestic distribution). Moreover, 68 countries (Canada is not among them) have curbed exports of personal protective equipment (PPE) or medicine during this crucial fight against COVID-19 by the front-line workers. A lack of supply and surging demands have sent prices soaring for all the PPE as well as air cargo rates [11]. In spite of these challenges the Canadian government is taking steps to get its PPE supply from China so that healthcare workers and vulnerable citizens are not put at risk.

## Conclusion

The recent emergence of COVID-19 poses a serious threat to human health and is currently causing significant social and economic disruption globally. There still remain certain challenges faced by Canada especially with the extensive land borders facilitating people crossing the borders by road, dependence on cross border road transport of goods from USA and also because of the economic downturn resulting in impacts on supply chains, commodity prices and global financial markets. There is a strong need for collective action by all key stakeholders through a multi-pronged approach to mitigate, prevent and fight against such health security threats (now and in future) through global health diplomacy. It must be remembered that enhancing the spirit of globalization, solidarity and recognizing the needs of developing countries is a more humane response than any nation pursuing a self-interested agenda which is not likely to leave a good legacy in global health or international relations.

## Data Availability

Not applicable.

## Abbreviations

2019-nCoV, or COVID-19, or SARS-CoV-2: Novel coronavirus
EID: Emerging Infectious Diseases
GHD: Global Health Diplomacy
GHS: Global Health Security
PPE: Personal protective equipment
PHEIC: Public Health Emergency of International Concern
WHO: World Health Organization
